# Chitosan (Nano)formulations as Therapeutic Tools for Neurodegenerative Diseases: A Comprehensive Review

**DOI:** 10.3390/polym17212838

**Published:** 2025-10-24

**Authors:** Adriana C. C. Gomes, Adelaide Almeida, Carmen S. R. Freire, Bárbara Leite Ferreira

**Affiliations:** 1 CICECO—Aveiro Institute of Materials, Department of Chemistry, University of Aveiro, 3810-193 Aveiro, Portugal; adrianagomes@ua.pt; 2 CESAM—Centre for Environmental and Marine Studies, Department of Biology, University of Aveiro, 3810-193 Aveiro, Portugal; aalmeida@ua.pt; 3 INSIGHT—Piaget Research Center for Ecological Human Development, 1950-157 Lisboa, Portugal

**Keywords:** chitosan, neurodegenerative diseases, nanoformulations, drug delivery, therapeutic tools

## Abstract

According to the World Health Organization, Alzheimer’s disease and other forms of dementia were the seventh leading cause of death in 2021. The prevalence of these disorders is predictable to increase with life expectancy, and their control is hampered by several factors, including late diagnosis due to the lack of specific biomarkers and the absence of disease-modifying treatments, as currently available therapies can only lighten some of the symptoms. Nanotechnology could be the key to overcoming some of the limitations associated with neurodegenerative diseases, as nanomaterials have excellent properties compared to their bulk counterparts and can be used as drug delivery systems, diagnostic tools and platforms for tissue regeneration. Chitosan is a biopolymer with numerous properties that impart it with great potential for biomedical applications, in particular its ability to cross the blood–brain barrier and its versatility in nanoscale design. In this context, the aim of this review is to provide an in-depth analysis of the latest developments and future opportunities for chitosan (nano)formulations for the treatment and management of neurodegenerative diseases.

## 1. Introduction

Neurodegenerative diseases are characterized by a gradual loss of nerve cells in the central or peripheral nervous system and can have various consequences, such as cognitive impairment, motor dysfunction, and even death [1,2]. According to the World Health Organization, Alzheimer’s disease (AD) and other forms of dementia were the seventh leading cause of death, causing 1.8 million deaths in 2021. In addition, this number has risen dramatically, almost quadrupling since 2000. In high-income countries, these disorders are the fourth leading cause of death and are on track to become one of the top three [3]. As the likelihood of developing these pathologies rises with age, their prevalence is projected to increase dramatically as life expectancy continues to grow. Furthermore, currently available therapies can only relieve some of the symptoms, but the progression of neurodegenerative diseases cannot be halted or even (effectively) slowed down. Therefore, there is an urgent need to not only understand and prevent their causes but also work towards their early detection, management and treatment [1,2].

Nanotechnology has become a ground-breaking technology for the production of materials at the nanoscale due to their practical applications in a variety of fields [4]. According to the common definition, a nanomaterial (e.g., nanofiber, nanotube or nanoparticle) has at least one dimension between 1 and 100 nm. However, many authors suggest that this definition needs to be revised as it is not scientifically proven and could limit the classification of nanomaterials, as in some cases, materials larger than 100 nm also exhibit nanoscale properties that are significantly different from their bulk counterparts [5,6]. These properties include a large surface area compared to volume, high flexibility in manipulation and functionalization, and the ability to circulate in the bloodstream over a long period of time and overcome cellular and/or tissue barriers. Therefore, nanomaterials are very versatile and can be used in distinct biomedical applications, such as drug delivery systems, contrast agents, diagnostic tools and also as platforms for tissue engineering and regeneration [4].

**Figure 1:**
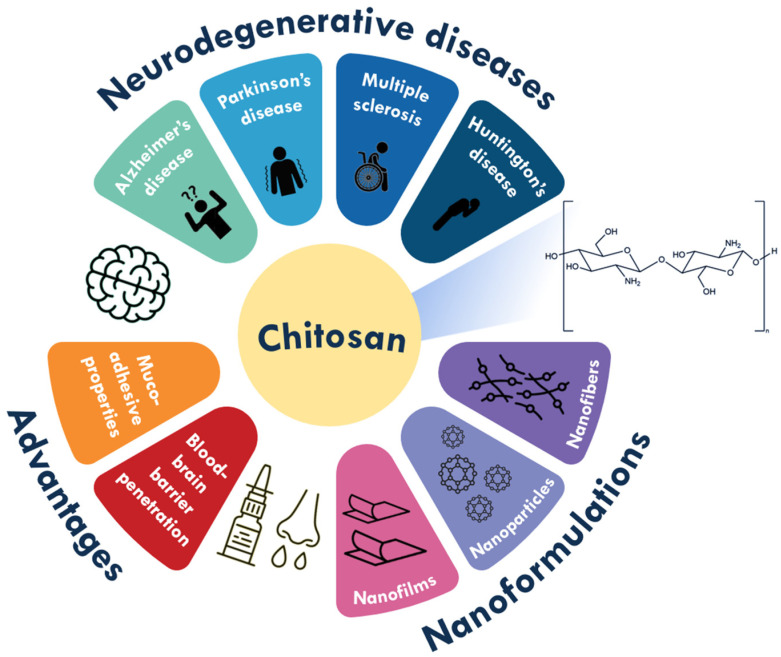
Schematic representation of chitosan (nano)formulations and their advantages as therapeutic tools for neurodegenerative diseases.

In particular, biopolymer-based nanomaterials have additional important properties such as biodegradability and biocompatibility, which give them great potential for biomedical endeavours, including drug delivery [7]. Chitosan is a polysaccharide that, in addition to the aforementioned advantages, has other properties such as biological functionalities like antimicrobial activity. Furthermore, chitosan has excellent mucoadhesive properties and can improve the permeability of drugs through the blood–brain barrier (BBB), which is why it has been extensively studied for the delivery of drugs to the brain, especially by intranasal administration [8]. Although recent reviews emphasize the role of chitosan in neurodegenerative diseases (e.g., [9]) and its use in brain drug delivery more broadly (e.g., [10]), comprehensive reviews dedicated exclusively to chitosan (nano)formulations for neurodegenerative diseases remain scarce. This review aims to refine and synthesize the existing evidence, highlighting their therapeutic potential for the management and treatment of these disorders (Figure 1).

## 2. Neurodegenerative Diseases

**Figure 2:**
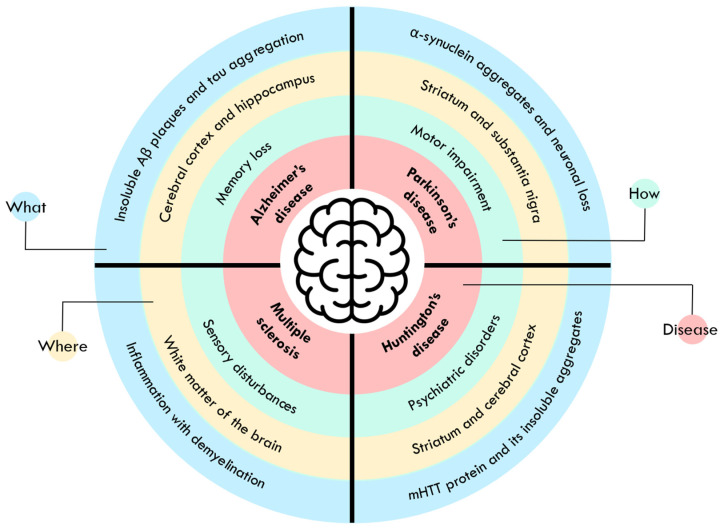
Overview of the main characteristics of neurodegenerative diseases, in particular Alzheimer’s disease, Parkinson’s disease, multiple sclerosis and Huntington’s disease.

Neurodegenerative diseases occur when neurons lose their functionality and eventually die [11,12]. Their clinical features depend not only on the specific areas where neuronal loss occurs, but also on genetic, metabolic and environmental factors [1,13]. Neurodegenerative disorders are age-dependent, and their clinical manifestation usually occurs late in life, even if progressive neuronal degeneration begins decades earlier. However, early diagnosis is hampered by the lack of specific biomarkers [12,13]. Furthermore, while there are therapies to reduce the side effects, there are no treatments that could cure or slow the progression of these diseases. This could be related to the restrictions imposed by the BBB, blood-cerebrospinal fluid barrier and P-glycoproteins, which prevent the penetration of drugs and cause side effects [11]. Neurodegenerative diseases comprise a large and diverse group of disorders, but in this review the focus will be placed on the most common ones, namely AD, Parkinson’s disease (PD), multiple sclerosis (MS) and Huntington’s disease (HD). Information on these disorders is discussed in the following subsections and illustrated in Figure 2.

### 2.1. Alzheimer’s Disease

AD is usually first detected in the frontal and temporal lobes and then spreads to other areas of the neocortex, affecting large parts of the cerebral cortex and hippocampus. It is characterized by the extracellular accumulation of insoluble amyloid-β (Aβ) plaques and the intracellular aggregation of tau protein in neurofibrillary tangles in the neurons [14,15]. There are two types of AD: sporadic or dominantly inherited. Sporadic AD is the most common form and is caused by a failure of the excretion mechanisms of the Aβ peptide from the brain tissue. Dominantly inherited AD, on the other hand, is characterized by pathogenic mutations in the genes encoding the amyloid precursor protein (APP) and the enzymes presenilin 1 and 2 (PS1 and PS2). The Aβ peptide is formed by the proteolytic cleavage of APP caused by PS1 and PS2, so mutations in the genes encoding these proteins often lead to an overproduction of the Aβ peptide [14,16].

The pathology of AD causes memory and cognitive impairment in a wide range of severity, from mild cognitive impairment, where cognitive abilities are not fully impaired, to dementia, where the degree of cognitive impairment severely interferes with the life of the patient [17]. Typically, episodic and semantic memory are affected, i.e., difficulty remembering recent events and facts, as well as linguistic, visuospatial and executive functions. It can also lead to impaired consciousness and neuropsychiatric disturbances, including depression, hallucinations and personality changes [1].

Cerebrospinal fluid biomarkers, including the decrease in Aβ_42_ (or Aβ_42_ normalized to Aβ_40_ or total tau (t-tau)) and the increase in phosphorylated tau (p-tau181), are useful diagnostic and monitoring indicators that can be used to predict the disease and study the effect of drugs in clinical trials [14,15,17]. There is currently no cure for AD, and available treatments are tailored to individual circumstances and attempt to alleviate symptoms. For example, cholinesterase inhibitors are used to delay the progression of symptoms in patients with moderate and severe dementia, but they cause adverse effects and have no impact on the progression of the disease [17].

### 2.2. Parkinson’s Disease

Patients with PD exhibit abnormal intraneuronal protein aggregates of α-synuclein, so-called Lewis bodies, and neuronal loss in certain areas of the substantia nigra [18]. The formation of Lewis bodies initially occurs in cholinergic and monoaminergic brainstem neurons and in neurons of the olfactory system but eventually spreads to the limbic and neocortical brain regions. In addition, a deficiency of dopamine and other neurotransmitters such as acetylcholine, serotonin and noradrenaline is also a feature of PD [19]. There are two forms of this disease: the genetically inherited form, either autosomal dominant or recessive, and the sporadic form, which is the most common and is thought to be caused mainly by gene-environment interactions [20]. Although various genes can be involved in inherited PD, the most common mutations occur in the genes for leucine-rich repeat kinase 2 (LRRK2), glucocerebrosidase (GBA) and the gene for α-synuclein (SNCA) [21,22].

The pathology of PD can be defined as a motor disorder as it causes motor symptoms such as resting tremor, stiffness, gait imbalance, and bradykinesia, which can be defined as a slowing of movement and decrease in amplitude or speed. However, it also causes non-motor effects such as cognitive impairment and dementia, psychiatric disorders such as depression and anxiety, sleep disturbances, and autonomic dysfunctions such as constipation, gastrointestinal motility problems and urinary symptoms. In fact, the worsening of non-motor symptoms increases as the disease progresses, impacting significantly patients’ quality of life [1,18].

The diagnosis of PD is largely delayed, as it is usually related to the assessment of motor impairment. Therefore, there is a need for specific and sensitive biomarkers, not only to detect the disease early but also to assess its progression [11,18]. Treatment is purely symptomatic and mainly focuses on pharmacological dopamine substitution, which includes levodopa or dopamine agonists. However, these drugs cause severe long-term complications, which is why great efforts are being made to find disease-modifying treatments [20,22].

### 2.3. Multiple Sclerosis

MS is an immune-mediated chronic inflammatory and neurodegenerative disease of the central nervous system (CNS) caused by a malfunction of the immune system. Although the exact cause of MS is not yet known, several factors such as vitamin D deficiency, smoking and obesity have been shown to contribute to the development of the disease [23]. The predominant features of this pathology are inflammation with demyelination and astroglial proliferation as well as neurodegeneration [24]. These lesions are caused by peripheral immune cells, mainly T and B lymphocytes and macrophages, and occur mainly in regions of the white matter of the brain, optic nerve and spinal cord, but can also be found in the intracortical and deep grey matter of the brain. The inflammatory demyelination and impaired healing eventually lead to permanent neurodegeneration and clinical disability [25].

In general, the pathology of MS can take two courses: relapsing-remitting or progressive. In the relapsing-remitting course, discrete episodes of neurological dysfunction typically occur, accompanied by inflammatory lesions with partial or complete regression. The progressive course, as the name implies, is the progression of the relapsing-remitting course over time, with gradual worsening and increasing clinical disability, but with less frequent inflammatory activity [24,25]. The disease causes a plethora of symptoms that vary depending on the location and severity of the lesions in the CNS, including sensory disturbances, unilateral optic neuritis, limb weakness, paroxysmal symptoms, fatigue, heat sensitivity, depression and cognitive impairment [23,24].

The diagnosis requires objective evidence of inflammatory CNS lesions, with the disease often occurring at multiple sites and multiple symptomatic events occurring within a given period of time. Magnetic resonance imaging (MRI) and cerebrospinal fluid analysis usually support the diagnosis [24]. However, due to the heterogeneity of the disease, it is very important to define molecular biomarkers to facilitate diagnosis and prognosis and to enable the evaluation of therapies, as reviewed by [26]. Current management strategies focus on treating acute attacks, alleviating symptoms and reducing biological activity through disease-modifying therapies. Although incredible progress has been made in the treatment of MS, leading to almost complete control of focal brain inflammation, this is not the case for the neurodegenerative component of the disease, for which an effective treatment is urgently needed [24].

### 2.4. Huntington’s Disease

HD is a dominantly inherited pathology caused by a CAG trinucleotide repeat expansion in the huntingtin (HTT) gene, leading to the formation of a mutant huntingtin (mHTT) protein containing an abnormally long polyglutamine sequence [27,28]. The mHTT protein tends to misfold and subsequently forms insoluble aggregates that accumulate and lead to neuronal dysfunction and apoptosis, eventually causing severe atrophy of the affected brain areas. Initially, these harmful aggregates appear to primarily affect the striatum, which is the neuropathological hallmark of HD, but atrophy is also observed in the cerebral cortex, cerebellum, hypothalamus and hippocampus [29].

HD causes a triad of symptoms. These include psychiatric disorders such as depression and anxiety, cognitive impairment with a decrease in attention, motivation, insight, problem-solving, and executive functions, behavioural symptoms such as aggressiveness, and motor dysfunction, typically characterized by excessive and involuntary movements, incoordination and bradykinesia [1]. As it is a progressive disorder, patients usually remain independent in the early stages but eventually require assistance due to worsening symptoms. In the later stages of the disease, dementia and cognitive impairment are severe. Unfortunately, death occurs a median of 18 years after the onset of symptoms, with infections, particularly aspiration pneumonia, being the most common cause of death [29].

The diagnosis of HD is based on clinical assessment, family history and, in most cases, genetic testing for the presence of the CAG expansion in HTT [30]. In addition, changes in cerebrospinal fluid and serum biomarkers, such as the presence of neurofilament light protein, which is released upon neuronal damage, are among the earliest detectable changes in HD and can predict the onset of the disease and track its progression. Although several clinical trials specifically targeting the disease are currently underway, current treatments for HD focus on symptom relief and are not disease-modifying treatments, i.e., they cannot halt or slow its progression [27].

## 3. Chitosan (Nano)formulations for Neurodegenerative Diseases

Chitosan can be obtained from chitin, the second most abundant polysaccharide in nature, which is found in the crustaceans’ shells, insects’ wings and even in some microorganisms, especially fungi [31]. Chitosan is a linear polysaccharide obtained by deacetylation of chitin via deacetylase hydrolysis or under alkaline conditions [8]. It consists of D-glucosamine and, to a lesser extent, N-acetyl-D-glucosamine units that are randomly β-(1→4)-linked [31]. Chitosan can establish inter- or intramolecular hydrogen bonds due to the hydroxyl and primary amino groups along its polymer backbone, and can also form electrostatic interactions in acidic media, having a polycationic character [32].

Chitosan has several properties that give it great potential for numerous biomedical applications, such as drug delivery and tissue engineering. These properties include biocompatibility, biodegradability, biological activity, such as antimicrobial effects, great versatility as it can be used in many forms (nanoparticles, nanofibers, nanogels), hydrophilic nature, good permeation effect, and mucoadhesion [31,32]. However, chitosan presents also some limitations, particularly its poor mechanical properties under wet conditions and its low solubility at pH values greater than 7. To overcome these drawbacks, various strategies have been reported, primarily involving its combination with other materials and its functionalization. These approaches can enhance solubility, reduce side-effects, improve drug loading and cellular uptake, and allow better control of drug release during administration [8,31,32].

The use of chitosan in neurodegenerative diseases is favoured by its ability to penetrate the BBB. The BBB separates the bloodstream and the CNS and mediates what passes through, e.g., nutrients and hormones, and what does not, e.g., external substances such as therapeutic molecules [31]. Chitosan interacts electrostatically with the negatively charged components of the tight junctions of the BBB, such as proteins and phospholipids, increasing their permeability and allowing larger molecules to pass through [8,32,33]. In addition, due to their small size, nanomaterials have easy access to pathways such as endocytosis, adsorptive-mediated transcytosis and paracellular pathways, which facilitates their penetration into the narrow spaces of the BBB [32,33]. On the other hand, chitosan and its derivatives have been reported to be neuroprotective in neurodegenerative diseases through various mechanisms such as antioxidant, anti-neuroinflammatory, anti-apoptotic and anti-excitotoxic effects, as described by Hao and co-workers [34].

To the best of our knowledge, Table 1 lists all examples of chitosan (nano)formulations for the management and treatment of neurodegenerative diseases [35,36,37,38,39,40,41,42,43,44,45,46,47,48], in particular AD [49,50,51,52,53,54,55,56,57,58,59,60,61,62,63,64,65,66,67,68,69,70,71,72,73,74,75], PD [76,77,78,79,80,81,82,83,84,85,86,87,88,89,90,91,92,93], MS [94,95,96,97,98,99,100,101,102,103] and HD [104]. These reports started around 2010 and have gained momentum in recent years, confirming their potential as a possible successful tool in the fight against these disorders. Most of the chitosan (nano)formulations described in the literature are nanoparticles (NPs), although other (nano)systems, such as nanofibers and nanofilms [56], nanocapsules [41,47,72,90], and nanogels [36,98] have been reported. The (nano)formulations are mostly used as drug delivery systems to manage these diseases, especially AD. In the following sections, some of the examples are described in more detail, grouped according to the type of pharmaceutical agent used in the drug delivery systems, e.g., by medicines currently in use, other drugs with neuroprotective potential, natural compounds with biological activities of interest and even RNA.

### 3.1. Alzheimer’s Disease

**Figure 3:**
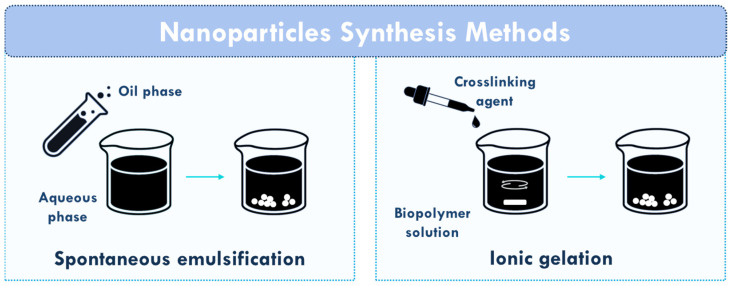
Schematic representation of the most commonly used methodologies for the synthesis of chitosan NPs for neurodegenerative diseases: spontaneous emulsification, in which two immiscible phases (generally an oil and an aqueous phase) spontaneously form NPs due to interfacial tension differences; and ionic gelation, in which oppositely charged polymers or ions interact electrostatically, leading to instantaneous NP formation through ionic crosslinking.

The most commonly used drugs for AD are inhibitors of the enzyme acetylcholinesterase to manage the symptoms [105]. The first studies on the use of chitosan nanosystems for AD management involved the incorporation of the acetylcholinesterase inhibitors tacrine [50] and rivastigmine [51] into chitosan NPs with particle sizes of 41 ± 7 nm and 47 ± 4 nm, respectively. The NPs were prepared by spontaneous emulsification (Figure 3) and exhibited a loading capacity of up to 13.37 ± 0.23% (tacrine) and 14.70 ± 0.17 *w*/*w* (rivastigmine), which varied with the drug/polymer ratio. In vitro release studies in phosphate-buffered solution (PBS) at a pH of 7.4 showed a burst effect within 30 min and up to 12 h, and the cumulative percentage release of tacrine from the NPs was up to 94.64 ± 0.84%, while for rivastigmine it was up to 97.25 ± 0.83%, both depending on the drug/polymer ratio. The NPs were also coated with polysorbate 80, which slightly reduced drug loading (10.86 ± 0.30% for tacrine and 11.51 ± 0.32 *w*/*w* for rivastigmine) and drug release (89.31 ± 1.48% for tacrine and 93.49 ± 1.53% for rivastigmine). Biodistribution studies in rats showed that the NPs altered the distribution pattern of the drug, with higher concentrations in the liver, spleen and lungs, and lower concentrations in the kidneys, which was further reduced in the polysorbate 80-coated NPs.

The acetylcholinesterase inhibitor galantamine was also encapsulated into chitosan NPs (diameter 190 ± 1.16 nm), which showed an encapsulation efficiency of 23.34%, a loading capacity of 9.86%, and a prolonged release of the drug (58.07 ± 6.67% after 72 h in distilled water with a pH between 6.5 and 7) [54]. The animal model studies confirmed that the NPs successfully reached various brain regions (olfactory bulb, hippocampus, orbitofrontal and parietal cortex) shortly after intranasal administration, suggesting their potential as a delivery system for AD management. Afterwards, galantamine/chitosan NPs were then further investigated following in vivo animal studies. It was found that the NPs did not adversely affect the pharmacological efficacy of the parent drug and did not show any clinical signs of toxicity or histopathological manifestations in the brain [55]. In addition, the NPs were observed in brain neurons and promoted a remarkable reduction in acetylcholinesterase levels and activity compared to the conventional oral and nasal formulations. Later, galantamine was loaded into chitosan NPs for nasal application for AD therapy [71]. The NPs (particle size of 240 ± 0.9 nm) were synthesized using the ionic gelation method (Figure 3) and galantamine was loaded during this process. Subsequently, some of the NPs were further coated with alginate (particle size of about 286 ± 3.1 nm). The chitosan NPs exhibited a galantamine loading efficiency of 67 ± 2.5%, while the alginate-coated NPs reached 70 ± 1.9%. The in vitro release studies in PBS (pH 7.2) at 37 °C demonstrated that the chitosan NPs provided a prolonged release of galantamine over a period of 8 h, while the alginate-coated NPs showed a faster (5 h) release of the drug, which can be explained by the pH of the medium, in which the alginate shell is highly soluble. In addition, both NPs were still stable after one year of storage at 5 °C.

It is recognised that the potential of a medicinal product extends beyond its intended or established primary use. Therefore, the urgent need for effective AD therapies is also being met by research into the effects of drugs originally intended for other diseases, such as diabetes or osteoporosis. For example, sitagliptin, an antidiabetic drug, was investigated for its effect on AD and showed promising results since cognitive functions were improved [106]. Recently, it was incorporated into chitosan NPs to investigate their potential for intranasal delivery to the brain [61]. Spherical NPs with a mean diameter of 188 ± 48.1 nm were formulated using the ionic gelation method, and the drug loading was in the range of 6.04 ± 2.3% to 7.87 ± 2.7% (*w*/*w*), depending on the drug/polymer ratio. In addition, the in vitro release studies (in PBS with a pH of 6.4) showed a cumulative percentage release of up to 73.77 ± 2.12% (*w*/*w*) over 24 h. Moreover, animal studies showed that the NPs increased brain sitagliptin concentrations by 5.07-fold after intranasal administration compared to the free drug. Similarly, chitosan–transfersulin nanovesicles (dispersed spherical shapes with a particle size of 138 ± 28.2 nm) were prepared by the film hydration method and evaluated as effective intranasal nanovesicles for the treatment of AD by mediating insulin transport to the brain [65]. Although the loading capacity of the drug was low (9.1%), the in vivo Morris water maze test showed that the treated rats exhibited a significant increase in learning and memory performance, as well as an improvement in cognitive function and neurogenesis in the hippocampus. In addition, in vivo optical imaging showed an improvement in intranasal insulin delivery due to a longer retention time and controlled release in the brain. The in vivo histopathological studies on different parts of the hippocampus also indicated a significant improvement in insulin delivery and therapeutic effect in the brain, as the treatment led to a marked increase in healthy neurons and pyramidal cells, showing a significant morphological improvement.

In the same context, alendronate, which is a drug commonly used to treat osteoporosis, also showed a protective effect against AD-like neuropathological changes in mice [107]. Chitosan NPs (135.75 ± 5.80 nm) were loaded with alendronate to be administered intranasally into the brain [62]. The drug loading was 30.92 ± 0.375% and the in vitro release studies (dialysis in PBS with a pH of 7.4) showed an initial burst release followed by sustained drug release (53.31 ± 1.05% at 2 h and 88.61 ± 2.11% at 24 h). The NPs achieved a high concentration in the brain of mice and a better pharmacokinetic profile than the alendronate solution (478.48 ± 37.24 ng/mL versus 294.05 ± 16.55 ng/mL). In addition, the NPs showed neuroprotective potential, improved memory and cholinergic deficits, and reduced the level of pathological markers in the hippocampus of animal models of AD (including Aβ, BACE-1 (APP processing), p-tau, and oxidative stress markers), suggesting high potential as a targeted system for AD treatment.

Furthermore, there is increasing interest in natural compounds commonly found in plants, vegetables and fruits that have biological activities of interest for biomedical applications. One example is ferulic acid, a natural bioactive phenolic compound with promising effects in neurodegenerative disorders. In the present context, ferulic acid was loaded into chitosan-coated solid lipid NPs for the effective management of AD [63]. The NPs (particle size of 185 nm) exhibited an entrapment efficiency of 51.18%, and the in vitro release studies in PBS at a pH of 6.5 showed that 64% of ferulic acid was released within 12 h, which was an improvement over the drug solution (19%) but more sustained compared to the non-coated NPs (72%). The ex vivo studies showed that the NPs exhibited increased mucoadhesion (from 6.88 to 8.55 N after coating with chitosan) and improved drug permeation through the nasal mucosa (35.49%). In addition, in vivo pharmacodynamic studies in rats following the Morris water maze test showed a reduction in escape latency time and a significant improvement in various biochemical parameters (4.4-fold increase in glutathione levels, 6.6-fold reduction in nitrite levels, 4.1-fold reduction in acetylcholinesterase activity, 5.2-fold improvement in superoxide dismutase activity and 6.9-fold increase in ferulic acid concentration in the brain of animals treated with the NPs compared to the free drug) and body weight gain, indicating a significant improvement in cognitive abilities.

**Figure 4:**
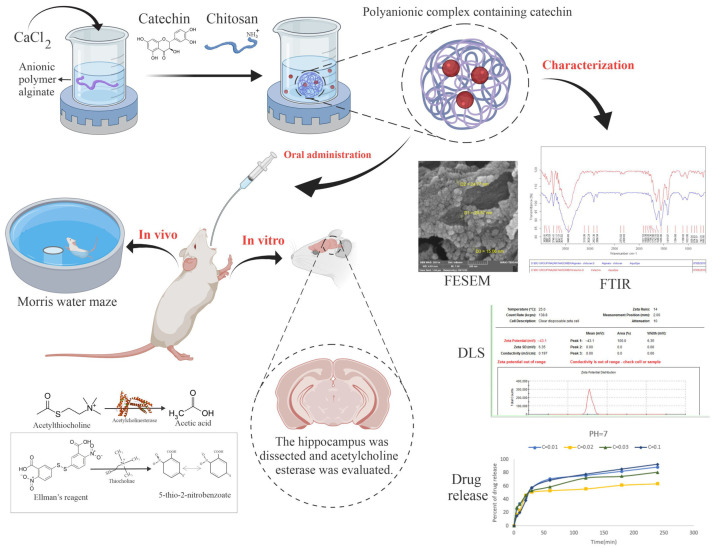
Overview of the study, which evaluates the protective effect of catechin-loaded chitosan-alginate NPs in an induced rat model of dementia. (DLS: dynamic light scattering; FESEM: field emission scanning electron microscopy; FTIR: Fourier transform infrared spectroscopy.) Reprinted from [75], an open access article distributed under the terms of the Creative Commons CC BY license (http://creativecommons.org/licenses/by/4.0/ (accessed on 12 July 2025)).

Another recent example involves catechin-loaded chitosan-alginate NPs, which is supported by growing preclinical evidence of catechins’ potential in AD prevention and treatment [75]. These spherical NPs, prepared by ionotropic gelation, presented a particle size predominantly between 40 and 45 nm and exhibited an entrapment efficacy of 60% (Figure 4). The in vitro release studies conducted in three buffers with varying pH values—KCl-HCl (pH = 1), acetate buffer (pH = 5), and Tris–HCl buffer (pH = 7)—proved that the NPs containing 0.1 mg of catechin were the most effective. This concentration allowed for a controlled release, with only 25% of catechin released at an acidic pH (mimicking digestive tract conditions) and 68% released at a pH of 7 (similar to blood circulation), promoting higher catechin availability for brain delivery. In addition, the nanocarriers lowered acetylcholinesterase activity (from 1.44 ± 0.25 to 0.96 ± 0.27 U/mg protein) and enhanced antioxidant defences as shown by an increase in catalase activity (from 43.05 ± 4.01 to 58.21 ± 3.99 U/g protein) in the brain of an AD rat model. Treatment with catechin-loaded chitosan-alginate NPs also led to improvements in spatial memory and learning in behavioural tests. These findings suggest that these nanocarriers could be a promising therapeutic approach to address the chemical and behavioural alterations associated with AD.

### 3.2. Parkinson’s Disease

Most drugs used for PD act to maintain dopamine levels in the brain, by either increasing them (levodopa and dopamine agonists) or preventing their decrease (monoamine oxidase-B, MAO-B, inhibitors such as rasagiline and selegiline). For example, rasagiline was encapsulated into chitosan-coated poly(lactic-co-glycolic) acid (PLGA) NPs for intranasal delivery to treat PD [80]. The NPs (particle size 122.38 ± 3.64 nm) were developed by the double emulsification and solvent evaporation technique. The encapsulation efficiency was 75.83 ± 3.76%, and the in vitro release profile obtained in PBS at a pH of 6.5 and 37 °C reached equilibrium after 14 h with a cumulative percentage of 81%. Ex vivo permeation studies on goat nasal mucosa, revealed a cumulative percentage of 81% after 24 h, compared to 19.5% for the rasagiline solution. In addition, the NPs showed better brain bioavailability when administered intranasally than intravenously (1465.22 ± 23.66 versus 381.36 ± 12.99 ng/mL), indicating their potential as a delivery system for the treatment of PD. In another study, selegiline was loaded into chitosan NPs prepared by ionic gelation with tripolyphosphate [83]. The obtained NPs had a size of 63.1 nm, a mucoadhesion of 65.4%, an entrapment efficiency of 74.8%, and a biphasic release profile of selegiline over 36 h, achieving a maximum plasma concentration of 52.71 ng/mL compared to 21.69 ng/mL of the drug solution. The in vivo animal studies showed a reversible effect on catalepsy and akinesia. In addition, a significant decrease in lipid peroxidation and nitrite concentration as well as an increase in the levels of reduced glutathione and catalase enzyme was demonstrated, which can be attributed to the antioxidant effect of selegiline.

In a different study, levodopa was loaded into carboxylated single-walled carbon nanotubes coated with various polymers, including chitosan [81]. The in vitro release studies showed that the cumulative release of levodopa was dependent on the pH of the medium, namely 45% at pH 7.4 and 22% at pH 4.8 in PBS at 37 °C. The cytotoxicity of the systems was investigated in vitro using an MTT assay on mouse embryonic 3T3 fibroblasts and it was found that these (nano)formulations did not affect cell viability even at the highest concentration tested (100 µg/mL), indicating better performance compared to the free drug, which showed a reduction in cell viability of more than 50% at a concentration above 50 µg/mL.

Dopamine agonists, such as pramipexole and ropinirole, have also been used in chitosan (nano)formulations for PD. For example, pramipexole was loaded by ionic gelation into chitosan NPs (292.5 ± 8.80 nm) with an entrapment efficiency of 91.25 ± 0.95%. The diffusion through the nasal mucosa of goats was 83.03 ± 3.48% after 24 h [82]. In vivo animal studies showed an improvement in locomotor activity and a reduction in motor deficits in the form of catalepsy. In addition, the antioxidant effect in the form of catalase activity (23.69 ± 2.96 versus 21.09 ± 3.21 units/mg protein) and dopamine levels (97.38 ± 3.91 versus 81.61 ± 4.44 ng/g tissue) were increased in the brain after intranasal treatment with the NPs compared to the drug solution. Subsequently, a chitosan-coated nanoemulsion, with spherical globules of 183.7 ± 5.2 nm, of ropinirole with nigella oil was developed [88]. The in vitro release and permeation studies exhibited a 2-fold and 3.4-fold enhancement, respectively, compared to the drug suspension. In addition, in vivo animal studies were conducted to evaluate the effect on the pharmacodynamics of the nanoemulsion. The results showed improved neurobehavioral function, a reduction in oxidative stress (glutathione levels were 3.80 ± 0.14 μmol/g tissue compared to 1.53 ± 0.09 μmol/g tissue of the drug suspension) and neuroinflammation by reducing NF-κB expression, as well as neuroprotection and reversal of histological aberrations. In addition, the nanoemulsion showed better targeting to the brain (36,180.99 ± 4582.14 versus 5680.10 ± 632.87 ng/mL) and thus exhibited higher bioavailability than the drug suspension. Similarly, ropinirole was incorporated into two (nano)formulations: a chitosan-alginate polyelectrolyte nanocomplex with a diameter of 402 ± 25 nm (encapsulation efficiency of 91 ± 5%) and chitosan-coated poly(ethyleneglycol)-b-poly(caprolactone) (PEG-b-PCL) nanocapsules with a diameter of 371 ± 20 nm (encapsulation efficiency of 87 ± 6%) [90]. The in vitro release studies in a simulated nasal electrolyte solution at pH 5.5 and 35 °C showed that both formulations exhibited a biphasic release profile with a burst in the first 2 h and a sustained release thereafter, with the nanocapsules showing a higher release of ropinirole. The cytotoxicity studies were performed on Raw 264.7 mouse macrophage cell line using the MTT assay. However, compared to the ropinirole solutions, which only reduced cell viability by up to 20% even at the highest concentration tested (1000 μg/mL), the (nano)formulations exhibited lower biocompatibility, with the IC50 of the nanocomplex and nanocapsules being 19.6 and 22.8 μg/mL, respectively. However, the radiolabelling technique revealed that both (nano)formulations improved the biodistribution and targeting of ropinirole in the brain (more than 2% compared to 0.93% radioactivity/g of ropinirole solution after 1 h), and that intranasal administration achieved higher targeting to the brain than intravenous administration, probably due to the mucoadhesive properties of chitosan.

**Figure 5:**
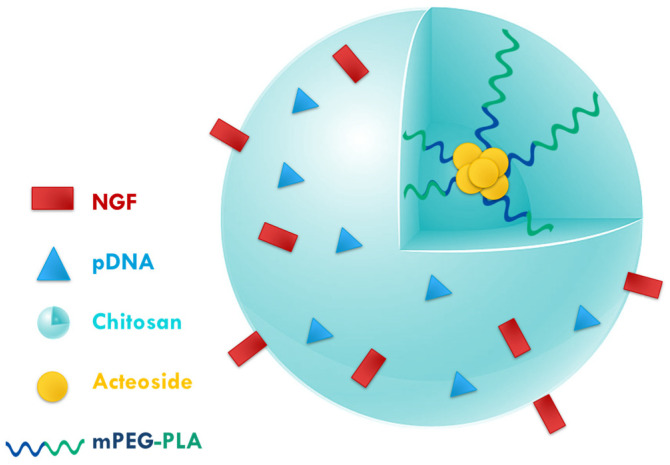
Schematic representation of chitosan-(PEG-PLA) NPs conjugated with nerve growth factor (NGF), acteoside and plasmid DNA (pDNA) (PEG: poly(ethyleneglycol); PLA: polylactic acid. Adapted from [85]).

Moreover, acteoside is a naturally occurring compound that has antioxidant and neuroprotective effects. This compound, along with the nerve growth factor (NGF) and plasmid DNA (pDNA), was conjugated with chitosan poly(ethyleneglycol)-polylactic acid (PEG-PLA) NPs (160 nm with positive charge) for PD therapy (Figure 5) [85]. The loading rates for pDNA were 0.49%, for NGF 0.73%, and for acteoside 6.64%. The in vitro studies showed that the NPs reduced the viability of PC12 + MPP+ (PD cell model) to 51%, but did not exhibit cytotoxicity in the absence of MPP+, indicating that the NPs are selectively toxic to damaged cells, which is a desirable property for targeting PD. In addition, the results showed that treatment with the NPs triggered a cell recovery effect in which the integrity of the nucleus was restored and the apoptotic rate was reduced from 50% to less than 10%, demonstrating a significant neuroprotective effect. FTIC fluorescence and Western blotting showed that α-syn aggregation decreased and NGF increased after treatment. Additionally, tests in an induced PD mouse model revealed an improvement in symptoms and consequently an improvement in behavioural disturbances. Furthermore, in vivo toxicity tests showed that the NPs caused no organ damage (brain, lung, liver, spleen, kidney and heart) and no differences in blood indices. These results were therefore very promising and the authors intend to further investigate the drug’s mechanism of action and conduct initial studies in humans.

### 3.3. Multiple Sclerosis

Dimethyl fumarate (DMF) and interferon-β (INFβ) are widely used in the management of MS and have also been combined with chitosan-based systems. Recently, INFβ was loaded into chitosan/sulfobutylether-β-cyclodextrin NPs for intranasal administration [96]. The obtained NPs presented hydrodynamic sizes between 202 and 280 nm and showed an association efficiency—calculated by subtracting the unbounded drug mass from the total drug mass added to the mixture before NPs formation—greater than 80%. Additionally, a functional assay measuring INFβ release, based on the expression of class I MHC proteins on L (tk-) mouse fibroblasts stimulated by IFN-β, showed that the NPs facilitate a slow and sustained release. The cell viability studies in both L(tk-) mouse fibroblasts and mouse splenocytes with NPs containing 25,000, 50,000 or 125,000 IU of IFN-β for 94 h, showed no toxicity, as cell viability was always above 70%. The in vivo biodistribution test of NPs loaded with fluorescent probes, administered intranasally, showed that the NPs could be detected in the brain and other organs such as the spinal cord and kidneys after 7 h, but no fluorescence was detectable in the brain after 24 h, in contrast to the liver, where it was largely detected. In addition, the therapeutic effect was investigated in vivo in a preclinical model of MS in mice. The spinal cord of treated mice exhibited fewer inflammatory cell foci and areas of demyelination, and the treatment also resulted in lower expression of antigen-presenting and costimulatory proteins on CD11b+ cells and reduced the activation of astrocytes and microglia. The results showed that intranasal administration of NPs is an efficient method to target the CNS and leads to a significant improvement of clinical symptoms, recovery from motor impairment and neurological damage, and control of neuroinflammation by regulating cell activation.

In addition, an oral film containing chitosan-alginate NPs loaded with DMF (with a particle size of 561 ± 53.05 nm) was developed to improve the drug bioavailability in the treatment of MS [97]. The NPs were prepared by ionotropic pre-gelation of the alginate core and subsequent polyelectrolyte complexation with chitosan. The resulting colloidal nanosuspension was incorporated into the optimized polymer matrix solution by a simple integration process and subsequently formed into films by solvent casting. The in vitro drug release profile of the film incorporating the NPs was significantly more sustained (18.39 ± 0.41% release in 30 min followed by sustained release up to 6 h) compared to the oral DMF film formulations (more than 80% within 15 min). In addition, an ex vivo permeation study on the buccal mucosa of pigs showed improved permeability of the NPs film, as it exhibited 56% higher permeability than the pure drug after 30 min. The in vivo pharmacokinetic study in animals confirmed that the NPs film had a 0.6-fold higher bioavailability compared to the conventional oral film formulation, even at a very low drug concentration (2 mg/film), which can be attributed to the mucoadhesive properties of chitosan. Similarly, DMF was incorporated into a chitosan nanogel (CN, particle size 111.33 ± 28.02 nm) and a platelet membrane-coated nanogel (PCCN, particle size 118.33 ± 16 nm) [98]. The entrapment efficiency and loading capacity of CN were 65.04 ± 3.53% and 6.47 ± 1.50% and of PCNN were 75.61 ± 4.84% and 8.12 ± 0.99%, respectively. The in vitro release study using dialysis in PBS at a pH of 7.4 and 37 °C showed that the drug was gradually released from the (nano)systems, with a release of about 90% after 30 h for both nanoplatforms. In contrast, the drug solution quickly reached equilibrium and was released almost 100% within 5 min. Pharmacokinetic studies in rats following intravenous administration show that the (nano)systems had a lower distribution in the peripheral tissues, reduced drug elimination, and achieved higher concentrations in the plasma (2078.43 ± 140.86 ng/mL (CN) and 2390.94 ± 614 ng/mL (PCCN)) and brain tissue (1754.52 ± 90.12 ng/mL (CN) and 2211.12 ± 180 ng/mL (PCCN)) compared to the free drug (1887.70 ± 277 and 854.24 ± 75.85 ng/mL, respectively). These effects may be partly attributed to the (nano)size of the drug carriers.

RNAs, such as small interfering RNAs (siRNAs) and microRNAs, are increasingly being researched as they have a promising potential to act on specific genetic mechanisms, modulate gene expression and offer innovative approaches for the treatment of various diseases. In the context of neurodegenerative diseases, siRNA has been used to block the expression of LINGO-1, a protein that suppresses remyelination. Thus, the effect of LINGO-1–directed siRNA-loaded chitosan NPs on demyelination and remyelination processes was investigated [95]. The in vivo studies in rats showed that intranasal administration of the NPs improved motor performance and coordination. In addition, signs of repair in the histopathological sections, higher expression of myelin basic protein (MBP) mRNA and protein, and lower levels of caspase-3 activity were observed compared to control rats, indicating neuroprotection and improvement of remyelination. Afterwards, microRNA-219a-5P (miR-219), which has been shown to be downregulated in demyelinating lesions, was also loaded into NPs containing chitosan, tragacanthic acid and glutathione to improve its delivery in the CNS [99]. The particle size of the NPs ranged from 227 to 558 nm. The results of in vivo studies in the cuprizone model of MS mice showed that treatment with the NPs led to an improvement in myelin sheaths, a reduction in inflammation and increased cell regeneration in the brain. In addition, an accumulation of miR-219 in the brain, its overexpression, an upregulation of crystallin alpha B and a downregulation of apolipoprotein E were demonstrated.

**Figure 6:**
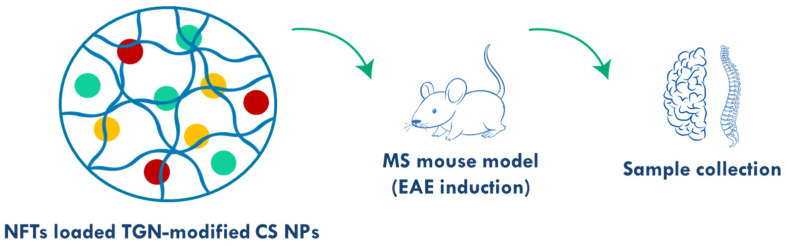
Overview of the study, including the TGN-modified chitosan NPs loaded with NFTs (CS: chitosan; EAE: experimental autoimmune encephalomyelitis; MS: multiple sclerosis; NFTs: neurotrophic factors. Adapted from [100]).

In a different vein, neurotrophic factors (NFTs) were loaded into TGN-modified chitosan NPs for the MS treatment and compared with mesenchymal stem cells (MSCs) and cerebrolysin administration [100]. The NPs were approximately 72 nm in size with a spherical shape and were prepared by adding tripolyphosphate to the previously prepared chitosan-PEG-TGN polymers (Figure 6). The in vivo studies in the MS mouse model revealed that the NPs provide neuroprotection, modulate immune responses (by reducing the expression of t-box transcription factor, matrix metalloproteinase and upregulating the expression of GATA3 and forkhead box p3 genes), and reduce inflammation (to 27% compared to 100% of the non-treated group), leading to an improvement in locomotor activity and a decrease in symptoms. The fluorescence images show that the NPs have high accessibility to the CNS and therefore have great potential for MS treatment.

### 3.4. Huntington’s Disease and Other Neurodegenerative Diseases

As far as we know, there are only two reports on chitosan (nano)formulations for HD therapy, both involving chitosan NPs loaded with anti-HTT siRNA. In the first study, published in 2020, different formulations of chitosan NPs were investigated by nasal administration into the brain in an in vivo mouse model of HD [104]. The NPs had an average hydrodynamic size between 104 and 205 nm, and the difference between them was caused by changes in the enrichment factor from 1.0 to 4.8, which allowed the loading of two different doses of siRNA, namely 1.2 and 5.8 nmol. Intranasal administration of the NPs resulted in the transport of anti-HTT siRNA from the nasal epithelium to various brain regions (olfactory bulb, cerebral cortex, corpus striatum and hippocampus), where the expression of HTT mRNA was reduced by at least 50%. The results showed that chitosan protects the siRNA from degradation on its way to the target site. However, for successful intranasal gene delivery, the NPs should be relatively small (100–160 nm) and have a high concentration of siRNA without damaging it. The other study, published one year later, investigated the kinetics of the reduction in HTT expression in an in vivo mouse model of HD after treatment with the previously described NPs [108]. To calculate the siRNA dosing regimens that cause long-term therapeutically significant gene knockdown, the authors developed a mathematical model based on measuring the magnitude and duration of the reduction in gene expression in specific brain tissues after single and repeated intranasal administration of the NPs (24 μL of NPs with 5.8 nmol of siRNA). It was found that the kinetics of HTT reduction differed in the distinct brain regions, with the olfactory bulb showing the fastest and strongest reduction. Furthermore, repeated administrations of siRNA-loaded NPs at shorter intervals but with longer duration resulted in a higher magnitude of cumulative HTT reduction. Specifically, two consecutive doses administered 6 h apart doubled the magnitude of the lowering effect compared to a single administration, and the duration of the knockdown effect also increased from 40 to 56 h. Thus, these NPs appear to be a promising therapeutic alternative to reduce the expression of mutant HTT.

All examples mentioned so far are reports aimed at a specific neurodegenerative disease. However, there are also studies that can be useful for these diseases in general, as they share common aspects. One example is the changes in glutamate levels, which are associated with various neurodegenerative diseases. For this reason, an amperometric glutamate (Glu) biosensor was developed for accurate in vitro and in vivo measurement of Glu levels [35]. The biosensor was based on the covalent immobilization of glutamate oxidase on carboxylated multi-walled carbon nanotubes, gold NPs (with a spherical shape and an average size of 20 nm) and a chitosan film deposited on the surface of a Au electrode. The biosensor showed an optimal response within 2 s at pH 7.5 and 35 °C, high sensitivity (155 nA/μM/cm^2^) and selectivity for glutamate, a low detection limit (1.6 μM) and good storage stability (up to 4 months). In addition, it was not affected by several serum substances, such as ascorbic acid, urea, triglycerides and glucose, in their physiological concentrations and had a wide linear range (5–500 μM).

Another example is the p38 MAPK signalling pathway, which is frequently dysregulated in neurodegenerative diseases and whose abnormal phosphorylation contributes to pathogenesis. In this context, chitosan nanocapsules loaded with a p38 MAPK inhibitor (PH797804) for intranasal administration were developed [41]. The nanocapsules exhibited a mean hydrodynamic diameter of 406.1 nm in water, an encapsulation efficiency of 21.5 ± 2.7%, and a final drug loading of 3.5 ± 0.5%. The results showed that the encapsulated inhibitor maintained its inhibitory effect and was able to penetrate into the brain tissue. Furthermore, the nanocapsules were able to effectively reduce the enzymatic activity of p38 MAPK in microglial and neuronal cells, both in vitro and ex vivo, as well as in a mouse model for AD. They could therefore be an effective strategy to reduce the excessive activity of this enzyme that occurs in several neurodegenerative diseases.

**Figure 7:**
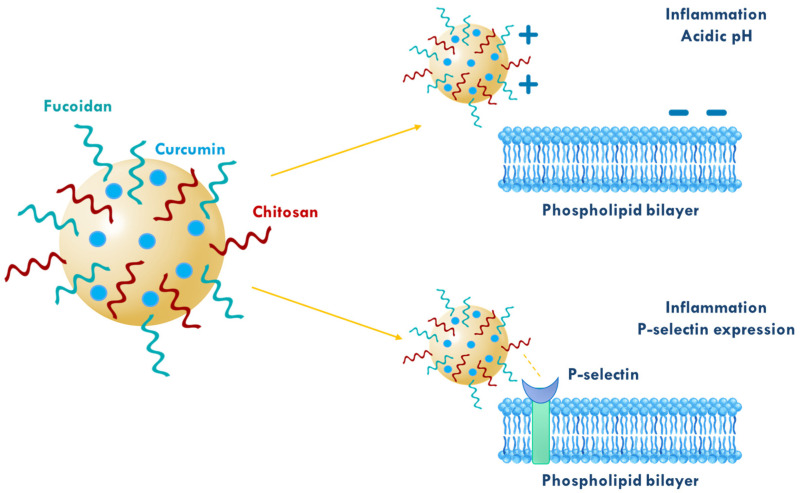
Schematic representation of curcumin-loaded chitosan/fucoidan nanocarriers with dual pH- and P-selectin-responsive properties that may enhance the reduction in brain inflammation (adapted from [43]).

In a different study, curcumin-loaded chitosan/fucoidan nanocarriers with a particle size of approximately 150 nm were designed aiming the inhibition of brain inflammation (Figure 7), as neuroinflammation is the initial symptom and a critical component in the progression of severe neurodegenerative diseases [43]. The encapsulation efficiency was increased from 45.7 ± 0.3% to 88.3 ± 0.8% and the loading content from 5.71 ± 0.04% to 9.96 ± 0.13% when the amount of chitosan in the curcumin/fucoidan/chitosan formulation was increased from 0.3/1.0/1.4 to 0.3/1.0/1.8 (weight ratio). The in vitro release study showed that curcumin was released sustainably over time, with an initial burst release (13.7% or 2.67 μg/mL in 10 min) followed by a slower path. The release rate was affected by pH, with a small amount of curcumin (less than 20%) released in an acidic environment. In vitro studies on a murine microglial cell line showed that the nanocarriers exhibited enhanced cellular uptake and inhibition of inflammation compared to free curcumin. Furthermore, in vivo studies in mice showed that intranasal administration of the nanocarriers led to an increased accumulation of curcumin in the brain (20.4% compared to 0.8% of free curcumin) and a better reduction in inflammation.

### 3.5. Mechanistic Insights into the Neuroprotective Effects of Chitosan

The neuroprotective properties of chitosan and its derivatives arise from multiple and complementary mechanisms that contribute to maintaining neuronal viability and function. Firstly, chitosan exhibits antioxidant activity by scavenging reactive oxygen species (ROS) and reactive nitrogen species (RNS), thereby reducing oxidative stress, a hallmark of neurodegenerative processes [109]. Its amino and hydroxyl groups can donate electrons and form complexes with metal ions such as Fe^2+^ and Cu^2+^, limiting Fenton-type reactions that generate free radicals [110]. In parallel, chitosan and its NPs modulate inflammatory responses, as evidenced by reduced levels of pro-inflammatory cytokines (TNF-α, IL-1β, IL-6) and downregulation of NF-κB and MAPK signalling pathways in glial cells [111]. These effects help mitigate microglial activation and neuronal apoptosis.

Another key mechanism is the ability of chitosan to enhance drug delivery across the BBB. The positive surface charge of chitosan enables electrostatic interactions with negatively charged tight-junction proteins and membrane components, leading to transient opening of paracellular pathways [112]. This facilitates the transport of encapsulated drugs to the brain, especially when combined with intranasal administration. Moreover, chitosan derivatives such as trimethyl chitosan and N-carboxymethyl chitosan have shown improved permeability and stability, further increasing drug bioavailability in the central nervous system [113].

Emerging evidence also suggests that chitosan may support neuroregeneration by promoting neuronal differentiation and neurite outgrowth, as well as by providing a biocompatible scaffold that mimics the extracellular matrix environment [114]. This property underlies its growing application in neural tissue engineering.

Despite these advances, important knowledge gaps remain. The precise molecular targets of chitosan within neuronal and glial cells are still poorly understood, and the relative contribution of its intrinsic bioactivity versus its carrier function has not been clearly established. Furthermore, most mechanistic studies rely on in vitro or short-term animal models, limiting the understanding of long-term neuroprotective and immunomodulatory effects. Future investigations combining molecular profiling, imaging, and computational modelling are needed to fully elucidate these mechanisms and optimise the therapeutic potential of chitosan-based systems.

### 3.6. Advances, Comparative Analysis, and Translational Challenges

Chitosan and its derivatives have attracted considerable interest as versatile, biocompatible, and biodegradable polymers for designing nanocarriers targeting neurodegenerative disorders. Their intrinsic mucoadhesive properties, cationic surface charge, and ability to transiently modulate tight junction permeability make them particularly effective for intranasal drug delivery, enabling direct nose-to-brain transport while bypassing the BBB. Over the past decade, numerous chitosan-based (nano)formulations (including NPs, nanocapsules, nanofibers, and core–shell systems) have been developed to enhance the targeted delivery, stability, and bioavailability of therapeutic agents, including conventional drugs, natural compounds, and nucleic acids, for the management of AD, PD, MS, HD, and related neurodegenerative conditions.

These systems have been investigated for a wide range of therapeutic agents, including acetylcholinesterase inhibitors, dopamine and its analogues, anti-amyloid peptides, siRNA, and natural antioxidants such as curcumin, lutein, and quercetin. Reported particle sizes range from 12 to 561 nm, a key determinant of pharmacokinetic behaviour: smaller nanoparticles (<150 nm) tend to cross the BBB more efficiently, providing rapid onset of action, while larger or polymer-coated formulations (>200 nm) enable sustained release and prolonged bioavailability. Chitosan blends with other polymers (e.g., chitosan–PLGA, chitosan–BSA, chitosan–PEG, and chitosan–PLA) combine mechanical stability with the mucoadhesive and permeability-enhancing properties of chitosan, achieving up to fivefold increases in brain drug concentration compared to free drugs.

Preclinical studies in AD and PD models have shown improved behavioural outcomes and reduced systemic toxicity, while chitosan-based nanocarriers loaded with immunomodulators or siRNA have demonstrated significant anti-inflammatory and remyelination effects in multiple sclerosis models. In HD, chitosan nanoparticles have effectively delivered anti-HTT siRNA via the nasal route, reducing mutant HTT expression by over 50%. Beyond therapeutic use, chitosan-based materials incorporating alginate, collagen, or fucoidan have shown neuroprotective effects under oxidative stress and excitotoxic conditions, while chitosan films integrated with gold or carbon nanostructures have been developed for biosensing applications, enabling rapid detection of neurotransmitters and neurotoxic biomarkers.

#### Critical Overview and Considerations for Clinical Translation

Despite promising preclinical performance, translational progress remains limited. Key challenges include ensuring reproducible large-scale synthesis, maintaining stability during storage, minimizing batch-to-batch variability, and addressing potential immunogenicity or mucosal irritation upon chronic administration. Most studies rely on rodent models, and interspecies differences in nasal anatomy and BBB permeability complicate extrapolation to humans. These limitations highlight the need for standardized protocols, advanced pharmacokinetic modelling, and well-designed clinical trials to evaluate safety, efficacy, and long-term outcomes.

Looking forward, successful clinical translation of chitosan-based nanocarriers requires a rigorous and integrated approach. Long-term toxicity studies, formulation stability optimization, and alignment with regulatory standards for nanomedicines are essential. Correlating physicochemical properties with biological performance will further enable rational design of systems with predictable in vivo behaviour. Additionally, combination therapies that integrate neuroprotective drugs, antioxidants, or gene-based therapeutics could maximize efficacy, while personalized nanomedicine approaches (tailoring formulation parameters according to patient-specific genetic, metabolic, or pathological profiles) may enhance therapeutic outcomes. Bridging these gaps will be fundamental to transforming promising laboratory prototypes into clinically viable therapies for neurodegenerative diseases.

## 4. Conclusions and Future Perspectives

### 4.1. Conclusions

This review focuses on chitosan (nano)formulations and their potential to fight neurodegenerative diseases, as they represent a promising option to overcome the limitations associated with these disorders. They can act as drug delivery systems capable of crossing the BBB and contribute to a more efficient treatment by improving neuroregeneration in damaged areas. As discussed, most chitosan (nano)formulations described in the literature in the context of neurodegenerative diseases are NPs designed for the effective delivery of several pharmacological agents. These include drugs currently prescribed for these diseases, drugs with neuroprotective effects, natural compounds with valuable biological activities and even RNA. In general, chitosan (nano)systems show greater potential compared to traditional formulations, particularly for intranasal administration. This approach led to higher drug concentrations in the brain, likely due to the mucoadhesive properties of chitosan, and resulted in neuroprotective effects along with improved behavioural and histological outcomes in animal disease models. However, it is challenging to compare the results because several studies either lack quantitative data or do not report or discuss them in detail. In addition, standardising experimental procedures would facilitate comparisons and correlations between studies, ultimately encouraging further research. Clinical trials would also be highly valuable at a later stage. Although current therapies do not modify the course of the disease, chitosan (nano)formulations appear to improve their bioavailability and reduce their toxicity, offering potential benefits for future treatments.

Addressing neurodegenerative diseases may also involve enhancing early diagnosis, which is currently hindered by the lack of specific biomarkers. Chitosan (nano)formulations are extremely versatile and could be used as diagnostic tools to detect biomarkers, such as Aβ42 and p-tau181 in the case of AD, or mHTT and neurofilament light proteins for HD. However, as far as we know, there are only two studies on chitosan (nano)platforms for the diagnosis of these diseases, namely a chitosan core coated with a biosensor (a polyamine-modified F(ab’) portion of IgG4.1) and chitosan-coated PLGA NPs conjugated with an anti-Aβ antibody, both targeting the cerebrovascular deposits of the AD amyloid protein [115,116]. These studies are crucial for advancing the detection of new biomarkers in the future and for evaluating the accuracy of these diagnostic tools.

On the other hand, neural tissue engineering offers the possibility of replacing damaged neural tissue with scaffolds that promote tissue regeneration. In addition, in vitro models that mimic the cellular and molecular interactions of the nervous system can also be developed, helping not only to understand the progression of neurodegenerative diseases but also to evaluate the effect of novel therapies and thus support their development. Chitosan (nano)formulations can also be used as scaffolds for neural tissue engineering, as demonstrated by a few research studies [117,118,119,120]. However, it is crucial to evaluate whether they are able to effectively promote the regeneration of damaged areas in vivo. Additionally, to the best of our knowledge, their potential as an integral part of in vitro disease models has not yet been explored and could help better understand the progression of neurodegenerative diseases and provide a platform for the evaluation and further development of new therapeutic approaches.

### 4.2. Future Directions and Clinical Outlook

Building upon these promising findings, future research should focus on advancing chitosan (nano)formulations from preclinical to clinical stages through carefully designed early-phase (I/II) trials. These studies should primarily evaluate safety, tolerability, biodistribution, and therapeutic efficacy, particularly for intranasal delivery systems that have shown superior brain-targeting potential. Incorporating neuroimaging and biomarker monitoring (such as Aβ, α-synuclein, or neurofilament light chains) could provide valuable insights into mechanisms of action and treatment responses. Combination strategies that integrate chitosan nanocarriers with neuroprotective drugs, antioxidants, or gene-based therapeutics may enhance synergistic effects and address disease complexity at multiple levels. Moreover, personalized nanomedicine approaches (optimizing formulation parameters according to patient-specific genetic, metabolic, or pathological profiles) could significantly improve therapeutic outcomes. Collaboration among materials scientists, neuroscientists, clinicians, and regulatory authorities will be essential to address challenges related to large-scale production, reproducibility, and long-term safety, ultimately enabling the successful translation of chitosan (nano)formulations from bench to bedside.

In conclusion, the research conducted to date strongly suggests the potential of chitosan (nano)systems for the management and treatment of neurodegenerative diseases. The prospect of discovering new biomarkers and developing disease-modifying therapies, combined with the remarkable properties of chitosan (nano)formulations, points to a promising future.

## Figures and Tables

**Table 1:** Examples of chitosan (nano)formulations as therapeutic and management tools for neurodegenerative diseases.

Disease	Nanoformulations (Size)	APIs	Results/Type of Administration	Ref.
**Alzheimer** **’** **s disease**	CS NPs (15 nm)	Amyloid-β peptide	High brain uptake efficiency (80.6% vs. 20.7% of the control group) and favourable immunogenicity (increased IgG against Aβ_42_)/IVA	[49]
	CS NPs (41 and 47 nm)	Tacrine and rivastigmine	Higher concentrations of the drug in the liver, spleen and lungs high in vitro cumulative percentage release (94.64% and 97.25%) up to 12 h/IVA	[50,51]
	CS NPs (between 100 and 200 nm)	Donepezil	Higher drug transport efficiency (191.39%) and direct transport percentage (1834.48%) compared to donepezil solution/INA	[52]
	CS NPs (249 nm)	Piperine	Relief of nasal irritation, no brain toxicity, a significant reduction in the drug dose (up to 20-fold) and improvement of cognitive functions/INA	[53]
	CS NPs (190 nm)	Galantamine	No clinical signs of toxicity or histopathological manifestations in the brain, remarkable reduction in acetylcholinesterase activity/INA	[54,55]
	CS NPs nanofilm (<250 nm) and CS/PVA nanofibers (<150 nm)	Donepezil	High drug loading capacity (>99%), the nanofiber showed higher bioavailability (31.6 ng/mL vs. 25.8 ng/mL)/OA	[56]
	CS and BSA NPs (144 nm)	Curcumin	Increased drug penetration through the BBB (60% vs. 30% of free curcumin) and accelerated phagocytosis of the Aβ peptide/NA	[57]
	CS/-PLGA NPs (between 100 and 120 nm)	-	Potent inhibitor of Aβ_1–42_: the amplitude of ThT fluorescence is reduced to 51% by CS-PLGA NPs and to 7% by CS NPs at 20 µg/mL/NA	[58]
	CS/PLGA core/shell NPs (<150 nm)	Lutein	Better crossing of the BBB (20.7% vs. 7.9%) and higher concentration in the brain (0.49% vs. 0.15%) compared to free lutein/INA	[59]
	CS-coated PLGA NPs (200 nm)	Curcumin	In vitro studies showed reduced cytotoxicity and decreased TNF-α and IL-6 levels to approximately 70 and 40%/INA	[60]
	CS NPs (188 nm)	Sitagliptin	Drug release up to 73.77% over 24 h, increased brain sitagliptin concentrations by 5.07-fold compared to the free drug/INA	[61]
	CS NPs (136 nm)	Alendronate	High concentration in the mice’s brain, better pharmacokinetic profile (478.48 vs. 294.05 ng/mL) than the alendronate solution/INA	[62]
	CS-coated solid lipid NPs (185 nm)	Ferulic acid	Increased mucoadhesion after coating (from 6.88 to 8.55 N), improved biochemical parameters and cognitive abilities of rats/OA and INA	[63]
	CS functionalized PLGA core/shell NPs (~200 nm)	Curcumin	Enhanced permeation through the nasal mucosa (79% vs. 40%) and BBB crossing (21% vs. 9%) compared to drug suspension/INA	[64]
	CS nanovesicles (138 nm)	Transfersulin	Significant improvement in memory performance, neurogenesis in the hippocampus, and intranasal insulin delivery to the brain/INA	[65]
	CS-embedded liposomes vs. CS-coated liposomes (142–277 nm)	*Centella asiatica* vs. Asiatic acid	Neuroprotective role and increased bioavailability (5.32 and 9.23 μg/mL) compared to asiatic acid (3.43 μg/mL)/OA	[66]
	CS NPs (413 nm)	Luteolin	Improved long-term memory by 85.7%, and 0.53- and 0.54-fold reduction in the levels of Aβ_1-42_ and Tau compared to the control/INA	[67]
	CS NPs (between 50 and 80 nm)	Tanshinone IIA	Prolonged life span, attenuation of symptoms, enhanced protective effect, and inhibition of oxidative stress in a *C. elegans* AD model/NA	[68]
	CS NPs (between 100 and 120 nm)	Chrysin	In vivo studies in zebrafish showed a reduction in amyloid β aggregates, neuronal death and generation of reactive oxygen species/OA	[69]
	CS nanocrystals (between 153 and 310 nm)	Memantine	Prolonged in vitro release and lower cytotoxicity than the drug solution in human nasal RPMI 2650 cells and goat nasal mucosa tissue/INA	[70]
	CS NPs and CS/ alginate-coated NPs (240 and 286 nm)	Galantamine	Loading efficiency of 67% and 70% and release of the drug over a period of 8 and 5 h, for CS NPs and CS alginate-coated NPs, respectively/INA	[71]
	CS-collagen nanocapsules	Magnoflorine	Inhibitory effects against oxidative stress, acetylcholinesterase, MDA and pro-inflammatory cytokines with increased SOD levels/IA	[72]
	CS NPs (161 nm)	Betanin	Significant antioxidant and anti-inflammatory activities, decreased acetylcholinesterase activity (IC_50_ 0.53 vs. 26 μg/mL of control)/NA	[73]
	CS NPs (131 nm)	Vinpocetine	Higher drug concentration in the brain (419 vs. 190 ng/mL) compared to oral administration, reducing systemic exposure/INA	[74]
	CS-alginate NPs (between 40 and 45 nm)	Catechin	In 1 h, 68% is released in pH 7, reduced acetylcholinesterase activity (0.96 vs. 1.44 U/mg protein)/OA	[75]
**Parkinson** **’** **s disease**	CS NPs (between 110 and 148 nm)	Dopamine	Reduced cytotoxicity and improved transport across the MDCKII-MDR1 cell line (fluorescence intensity increased 12-fold after 180 min)/IA	[76]
	CS NPs (161 nm)	Bromocriptine	Better reversal in catalepsy behaviour (48 s vs. 153 s) and enhanced brain concentration (0.03 vs. 0.01%/g) compared to drug solution/INA	[77]
	Zinc hydroxide/CS nanocarrier	Carbidopa	More controlled drug release in vitro (pH 7.4: 47% in 315 min vs. 82% in 255 min) than Zn hydroxide/NA	[78]
	CS-coated lipid carrier (137 nm)	GDNF	Protect PC-12 cells against 6-OHDA toxin, behavioural improvement in rats, improved density of TH+ fibres in the striatum by at least 40%/INA	[79]
	CS-coated PLGA NPs (122 nm)	Rasagiline	Improved release on goat nasal mucosa (81% vs. 20% of the drug solution) and better bioavailability in the brain/INA	[80]
	Carboxylated single-walled carbon nanotubes coated with CS	Levodopa	Drug release dependent on the pH (45% at pH 7.4 and 22% at pH 4.8), non-cytotoxic to mouse embryonic 3T3 fibroblasts at 100 µg/mL/NA	[81]
	CS NPs (293 nm)	Pramipexole	Improvement in locomotor activity and a reduction in motor deficits, increased antioxidant effect and dopamine levels in the brain/INA	[82]
	CS with tripolyphosphate NPs (63 nm)	Selegiline	Higher plasma concentration (52.71 ng/mL vs. 21.69 ng/mL of the drug solution), reversible effect on catalepsy and akinesia/INA	[83]
	CS NPs (220 nm)	-	Reduced rotenone-initiated cytotoxicity and apoptotic cell death (by 25 to 30%) probably due to antioxidant and anti-apoptotic properties/NA	[84]
	CS (PEG-PLA) NPs (160 nm)	Act, NGF and pDNA	Neuroprotective effect: reduced cell viability of the PC12 + MPP^+^ PD cell model to 51% but were not cytotoxic in the absence of MPP^+^/IA	[85]
	CS-coated PLGA NPs (468 nm)	Ropinirole hydrochloride	Increased drug permeation through sheep nasal mucosa by 3.22-fold compared to non-coated NPs, lower Raw 264.7 and PBMC viability/INA	[86]
	CS NPs (<100 nm)	Rotigotine	Increased brain concentration (61.72 vs. 36.74 ng/mL) and higher SOD enzyme levels (0.248 vs. 0.147 U/mL) compared to drug solution/INA	[87]
	CS-coated nanoemulsion (184 nm)	Ropinirole with nigella oil	Better targeting to the brain (36,181 vs. 5680 ng/mL of the drug suspension) and improved neurobehavioral function/INA	[88]
	CS NPs (~100 nm)	FTY720	1.3-fold increase in percentage cell survival against rotenone and induction of the PP2A-EzH2 mediated pSer129 α-Syn degradation/IVA	[89]
	CS-alginate polyelectrolyte nanocomplex (402 nm) and CS-coated (PEG-b-PCL) nanocapsules (371 nm)	Ropinirole	IC_50_ against Raw 264.7 mouse macrophage cell line of 19.6 (nanocomplex) and 22.8 (nanocapsules) μg/mL, improved targeting to the brain (more than 2% compared to 0.93% radioactivity/g of ropinirole solution)/INA	[90]
	Carboxymethyl CS NPs (292 and 459 nm)	Dopamine	Antioxidant activity, no cytotoxicity against neuroblastoma SH-SY5Y cells and increased cell uptake demonstrated in 40 to 66% of cells/INA	[91]
	CS-coated nanostructured lipid carriers (<200 nm)	Tanshinone IIA	Significant increase in GSH levels by 154.6% with 48% and 84.6% reduction in MDA and HO-1 levels compared to the control/INA	[92]
	CS NPs	Curcumin	Restored ATP production by 195.5% and mitigated oxidative stress (by reducing ROS and MDA levels by 43.05% and 56.89%, respectively)/OA	[93]
**Multiple sclerosis**	CS/PLA nanofibrous scaffold (diameter 100 nm)	PC12 cells	PC12 cells attach, grow, and differentiate into neural-like cells that further reduce symptoms, axonal damage and demyelination/NA	[94]
	CS NPs	siRNA	Improved motor performance and coordination, neuroprotection and improvement of remyelination/INA	[95]
	CS/sulfobutylether-β cyclodextrin NPs (between 202 and 280 nm)	INFβ	No toxicity towards L(tk−) mouse fibroblasts and mouse splenocytes, in vivo studies in a MS model in mice showed lower clinical symptoms/INA	[96]
	CS-alginate NPs (561 nm)	DMF	Sustained drug release (18% in 30 min vs. 80% in 15 min) and 0.6-fold higher bioavailability compared to an oral DMF film formulation/OA	[97]
	CS nanogel (111 nm) and platelet membrane-coated nanogel (118 nm)	DMF	Sustained drug release, in vivo pharmacokinetic studies in rats indicated higher plasma and brain concentrations compared to the free drug/IVA	[98]
	CS, tragacanthic acid and glutathione NPs (between 227 and 558 nm)	miR-219	Improved myelin sheaths, reduced inflammation and increased cell regeneration in the brain/IVA	[99]
	TGN-modified CS NPs (72 nm)	NFTs	Neuroprotection and reduced inflammation (to 27% compared to 100% of the non-treated group)/IA	[100]
	CS NPs (120 nm)	β-asarone and astragaloside IV	Reduced behavioural scores, suppressed inflammatory infiltration and astrocyte/microglial activation, and increased remyelination/INA	[101]
	CS/PEG NPs (240 nm)	Fluoxetine	Reduced anxiety, improved memory, increased BDNF levels, and reduced extent of demyelination, with no change in IGF-levels/OA	[102]
	Lactoferrin/CS double-coated oleosomes (220 nm)	Clobetasol propionate	Improved functions of mice, 2.3 folds increase in corpus callosum thickness, remyelination with 6.6 folds reduction in CP dose/INA	[103]
**Huntington** **’** **s disease**	CS NPs (between 104 and 205 nm)	anti-HTT siRNA	Reduced expression of HTT mRNA in the brain by at least 50%/INA	[104]
**Other neurodegenerative diseases**	Biosensor: carboxylated multi-walled carbon nanotubes, gold NPs, CS film, Au electrode	Glutamate oxidase	Response within 2 s at pH 7.5 and 35 °C, high sensitivity (155 nA/μM/cm^2^), low detection limit (1.6 μM) and wide linear range (5–500 μM)/NA	[35]
	CS-myristate nanogel (<50 nm)	-	Protection of neuroserpin from misfolding and aggregation/NA	[36]
	CS-alginate NPs	Quercetin	Neuroprotection in a model of H_2_O_2_-induced oxidative stress in neuroblastoma SH-SY5Y cells and of 6-OHDA in rat brain synaptosomes/NA	[37]
	CS-mangafodipir NPs (between 90 and 114 nm)	siRNA and dsDNA	Reduced GFP mRNA (by at least 50%) and RFP expression in multiple brain regions (e.g., cerebral cortex, hippocampus, and stratium)/INA	[38]
	CS-coated nanoemulsions (258 nm)	Rosmarinic acid	Sustained permeation through porcine nasal mucosa (47 vs. 132 µg cm^−2^ after 8 h) compared to the drug solution, non-cytotoxic to fibroblasts/INA	[39]
	CS NPs (between 300 and 400 nm)	Genistein	60% of the drug permeated through the nasal mucosa compared to none of the drug solution, and showed no cytotoxicity to PC12 cells/INA	[40]
	CS nanocapsules (406 nm)	p38 MAPK inhibitor	Reduced enzymatic activity of p38 MAPK in microglial and neuronal cells in vitro and *ex vivo*, as well as in a mouse model for AD/INA	[41]
	CS/carbon dots (144 nm)	Dopamine	In vitro drug release is pH dependent (60% at pH 4 and 4.5% at pH 7), not cytotoxic to IC-21 and SH-SY5Y cell lines/NA	[42]
	CS/fucoidan nanocarriers (150 nm)	Curcumin	In vivo studies in mice showed increased accumulation of curcumin in the brain (20.4% compared to 0.8% of free curcumin)/INA	[43]
	Nanospheres of covellite copper sulphide with CS (15 nm)	Dopamine	Photo-controlled drug release (from 6% to 50% of drug release in 5 h), non-cytotoxic to A549, L132 and SH-SY5Y cell lines at 100 μg/NA	[44]
	CS NPs (104 nm)	*Ginkgo Biloba* extract	Neuroprotective activity by increasing the viability of SY5Y cells from 60% to 92.3% (also higher than that of the free extract (83.9%))/NA	[45]
	CS/lecithin NPs (218 nm)	Statin	11-fold increase in drug permeation across a human cell model of the nasal epithelium, stronger suppression of pro-inflammatory signalling/INA	[46]
	CS–collagen nanocapsules (12 nm)	Magnoflorine	Good antioxidant potential (IC_50_ < 25 μg/mL), 85.50% viability of SH-SY5Y cells at 50 μg/mL and good acetylcholinesterase inhibitor (85.20%)/NA	[47]
	CDX-modified CS NPs (110 nm)	Fingolimod	Reduced INF-γ levels, decreased expression of TBX21, GATA3, FOXP3 and Rorc, efficient cellular uptake, and regulation inflammation/IA	[48]

6-OHDA: 6-hydroxydopamine; Act: acteoside; AD: Alzheimer’s disease; APIs: active pharmaceutical ingredients; BBB: blood-brain barrier; BDNF: brain-derived neurotrophic factor; BSA: bovine serum albumin; CAT: catalase; CP: clobetasol propionate; CS: chitosan; DMF: dimethyl fumarate; GDNF: glial cell-derived neurotrophic factor; GFP: green fluorescent protein; GSH: glutathione; HO-1: hemoxygenase-1; HTT: huntingtin; IA: intraperitoneal administration; IL-6: interleukin-6; INA: intranasal administration; INFβ: interferon-β; IFN-γ: interferon-γ; IGF: insulin-like growth factor; IVA: intravenous administration; MDA: malondialdehyde; MPP+: 1-methyl-4-phenylpyridinium; MS: multiple sclerosis; NA: not applicable; NFTs: neurotrophic factors; NGF: nerve growth factor; NPs: nanoparticles; OA: oral administration; PBMC: peripheral blood mononuclear cell; PCL: poly(caprolactone); PD: Parkinson’s disease; pDNA: plasmid DNA; PEG: polyethylene glycol; PLA: polylactic acid; PLGA: poly(lactic-co-glycolic) acid; RFP: red fluorescent protein; ROS: reactive oxygen species; SOD: superoxide dismutase; ThT: thioflavin T; TNF-α: tumour necrosis factor-α.

## Data Availability

No new data were created or analyzed in this study.

## Abbreviations

The following abbreviations are used in this manuscript:

6-OHDA	6-hydroxydopamine
Aβ	Amyloid-β
AD	Alzheimer’s disease
APIs	Active pharmaceutical ingredients
APP	Amyloid precursor protein
BBB	Blood–brain barrier
BDNF	Brain-derived neurotrophic factor
BSA	Bovine serum albumin
CAT	Catalase
CN	Chitosan nanogel
CNS	Central nervous system
CP	Clobetasol propionate
CS	Chitosan
DLS	Dynamic light scattering
DNA	Deoxyribonucleic acid
DMF	Dimethyl fumarate
FESEM	Field emission scanning electron microscopy
FTIR	Fourier transform infrared spectroscopy
GBA	Glucocerebrosidase gene
GDNF	Glial cell-derived neurotrophic factor
GFP	Green fluorescent protein
GSH	Glutathione
HD	Huntington’s disease
HO-1	Hemoxygenase-1
HTT	Huntingtin gene
IA	Intraperitoneal administration
IL-6	Interleukin-6
INA	Intranasal administration
INFβ	Interferon-β
IFN-γ	Interferon-γ
IGF	Insulin-like growth factor
IVA	Intravenous administration
LRRK2	Leucine-rich repeat kinase 2 gene
MAO-B	Monoamine oxidase-B
MBP	Myelin basic protein
MDA	Malondialdehyde
mHTT	Mutant huntingtin
MPP+	1-methyl-4-phenylpyridinium
MRI	Magnetic resonance imaging
MS	Multiple sclerosis
MSCs	Mesenchymal stem cells
NA	Not applicable
NFTs	Neurotrophic factors
NGF	Nerve growth factor
NPs	Nanoparticles
OA	Oral administration
PBS	Phosphate-buffered solution
PBMC	Peripheral blood mononuclear cells
PCCN	Platelet membrane-coated nanogel
PCL	Poly(caprolactone)
PD	Parkinson’s disease
pDNA	Plasmid DNA
PEG	Polyethylene glycol
PLA	Polylactic acid
PLGA	Poly(lactic-co-glycolic) acid
PS1	Presenilin 1
PS2	Presenilin 2
p-tau181	Phosphorylated tau
RFP	Red fluorescent protein
RNA	Ribonucleic Acid
ROS	Reactive oxygen species
siRNAs	Small interfering RNAs
SNCA	α-synuclein gene
SOD	Superoxide dismutase
ThT	Thioflavin T
TNF-α	Tumour necrosis factor-α
t-tau	Total tau
